# Effect of Particle Interactions on the Assembly of Drying Colloidal Mixtures

**DOI:** 10.1021/acs.langmuir.1c03144

**Published:** 2022-04-19

**Authors:** James D. Tinkler, Alberto Scacchi, Maialen Argaiz, Radmila Tomovska, Andrew J. Archer, Helen Willcock, Ignacio Martín-Fabiani

**Affiliations:** † Department of Materials, Loughborough University, Loughborough LE11 3TU, U.K.; ‡ Department of Chemistry and Materials Science, Aalto University, P.O. Box 16100, FI-00076 Aalto, Finland; § Department of Applied Physics, Aalto University, P.O. Box 11000, FI-00076 Aalto, Finland; ∥ POLYMAT and Departmento de Química Aplicada, Facultad de Ciencias Químicas, University of the Basque Country, UPV/EHU, Joxe Mari Korta Zentroa, Tolosa Hiribidea 72, Donostia-San Sebastian 20018, Spain; ⊥ Ikerbasque, Basque Foundation for Science, Maria Diaz de Haro 3, 48013 Bilbao, Spain; # Department of Mathematical Sciences and Interdisciplinary Centre for Mathematical Modelling, Loughborough University, Loughborough LE11 3TU, U.K.

## Abstract

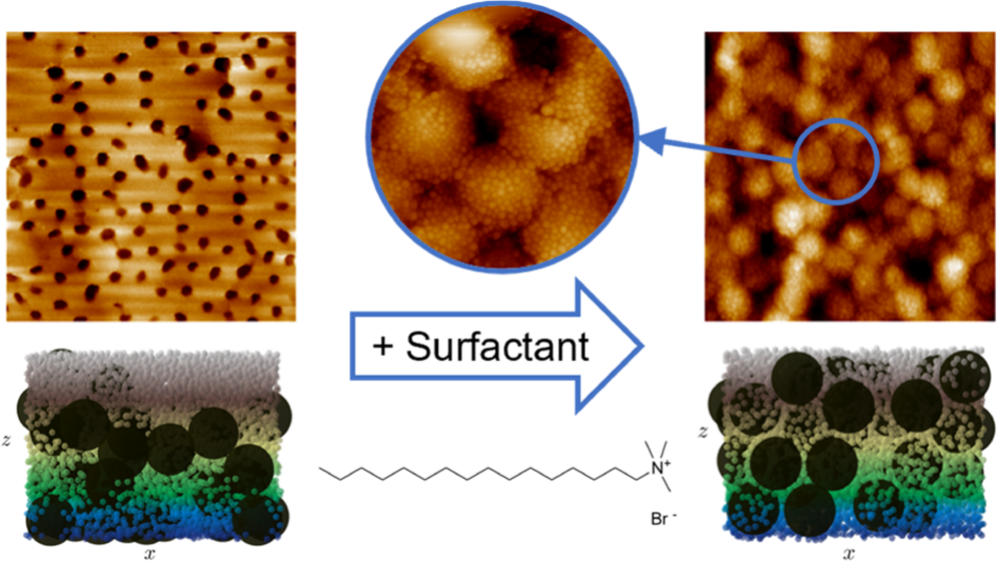

The effects of particle interactions on the size segregation and assembly of colloidal mixtures during drying were investigated. A cationic surfactant was added to a binary latex/silica colloidal dispersion that has been shown to self-stratify upon drying at room temperature. Atomic force microscopy was used to show that the change in particle interactions due to the presence of surfactants reduced the degree of stratification and, in some cases, suppressed the effect altogether. Colloidal dispersions containing higher surfactant concentrations can undergo a complete morphology change, resulting instead in the formation of armored particles consisting of latex particles coated with smaller silica nanoparticles. To further prove that armored particles are produced and that stratification is suppressed, cross-sectional images were produced with energy-dispersive X-ray spectroscopy and confocal fluorescence microscopy. The growth of armored particles was also measured using dynamic light scattering. To complement this research, Brownian dynamics simulations were used to model the drying. By tuning the particle interactions to make them more attractive, the simulations showed the presence of armored particles, and the size segregation process was hindered. The prevention of segregation also results in enhanced transparency of the colloidal films. Overall, this research proves that there is a link between particle interactions and size segregation in drying colloidal blends and provides a valuable tool to control the assembly of different film architectures using an extremely simple method.

## Introduction

Coatings can be produced by drying colloidal dispersions containing polymer particles.^1^ These dispersions are known as latexes and are commonly used in paints,^2^ inks,^3^ adhesives,^4^ and cosmetics.^5^ Dual-layered films, which can provide specific surface properties, are highly desirable for applications such as antibacterial coatings,^6^ abrasion resistant paints,^7^ antireflective coatings,^8^ and inkjet printing.^9^ In recent years, it has been shown that size segregation during drying of bimodal or polydisperse colloidal mixtures offers a promising method to produce stratified films containing two layers through a single-step process,^10^ reducing production time and cost compared with conventional multistep deposition methods.^11^

Vertical segregation can occur during the drying of bimodal colloidal mixtures as a result of diffusiophoresis^12^—the diffusion of particles along a concentration gradient caused by particles being swept up by the descending water/air interface. This affects larger particles more and can lead to small-on-top stratification. In some cases, superstructures have been observed at the top surface of stratified colloidal films.^6,7,13^ While diffusiophoresis is now widely accepted to be the main driving force for this stratification, the exact mechanisms leading to the formation of different superstructures at the film surface are not fully understood. In our previous research, we suggested that particle interactions (in particular the electrostatic repulsion between the same and different species) and the evaporation rate play a key role in the formation of such structures.^6,7^

The effects of particle charge on the vertical self-segregation of colloidal mixtures were first investigated by Nikiforow et al.^14^ They produced stratified films by drying latex blends containing a mixture of charged and neutral particles of the same size. The process still relies on initial particle accumulation at the drying front due to a lower rate of particle diffusion compared with evaporation (Péclet numbers greater than 1). The accumulation of particles at the film surface results in a concentration gradient, which, as described above, causes diffusiophoresis to drive them away from the surface again. However, the charged particles are subjected to an additional driving force due to the electrostatic repulsion between each other. This enhances the diffusiophoresis of only the charged particles, leading to an enrichment of neutral particles at the surface. It is also possible, depending on the size of the electric double layer, that this occurs due to the effective diameter of the charged particles being larger. This would cause the charged particles to be affected more by diffusiophoresis, resulting in a surface enrichment of neutral particles. Similar studies were also carried out by Atmuri et al.,^15^ who varied the charge of polymer colloids via pH and observed the effects on stratification. They also produced models of the drying process to demonstrate the effects of interactions between charged particles. It is worth noting that neither Nikiforow et al. nor Atmuri et al. consider the effects of attractive particle interactions on stratification.

Other than our own studies and those of Nikiforow et al. and Atmuri et al., there is very little research into the effects of electrostatic particle interactions on stratification. There has, however, been some intensive research into the effects on the coffee-ring effect. Noguera-Marín et al.^16^ showed that the particle diffusion due to charge repulsion was dominant enough to counteract the coffee-ring effect, with particles being driven away from the contact line. Further investigation showed that this mechanism was also dependent on the charge-mass ratio of the particles.^17^ Segregation between particles was observed due to greater charge-mass ratios resulting in stronger resistance to sedimentation. Anyfantakis et al.^18^ also attempted to suppress the coffee-ring effect via particle interactions. They introduced surfactants capable of bonding to the surfaces of colloidal particles, effectively altering their surface charge. They found that when the particle surface charge was sufficiently reduced, the particles became hydrophobic, attaining an affinity to the liquid–air interface. The particle accumulation at the air–water interface prevented capillary radial outward flow, which has a significant impact on the coffee-ring effect.

There are several other pieces of literature that focus on the effects of particle interactions on colloidal assembly, many of which utilize surfactants to alter the particle surface charges. For example, surfactants were used by Shevchenko et al.^19^ to show that altering particle charges can result in the formation of various different nanoparticle superlattice structures in binary colloidal dispersions. Bartlett and Campbell^20^ showed that particle charge and the resulting interactions resulted in the stabilization of colloidal superlattices that would be otherwise entropically unfavorable. Electrostatic particle interactions were also utilized by Hueckel et al. to control crystallization, producing ionic colloidal crystals in water.^21^

This research aims to better understand the influence of particle interactions on the stratification of binary colloidal dispersions and the formation of surface superstructures. In our previous work,^7^ we suggested that grid-like superstructures, observed at the surfaces of stratified colloidal coatings, may be a result of electrostatic interactions between large and small particles during drying. Here, we further investigate the effects of these interactions through experiments and simulations. We introduce cationic surfactants to binary colloidal systems, with the aim of controlling the particle surface charges, thereby altering the electrostatic interactions between particles. Using a wide range of experimental and modeling methods, we show that the addition of these surfactants has a significant effect on the final film assembly configuration. At first, the stratification effect is reduced, resulting in thinner layers of small particles at the film surface. Higher surfactant concentrations inhibit colloidal stratification completely, with armored particles being produced instead.

Herein, we prove the key role of particle interactions in the size segregation process in drying colloidal mixtures. We show that, by simply adding surfactants, the stratification can be repressed. We provide valuable results and insights that aid in controlling the architecture of the final dried films and, therefore, will help with the development of functional colloidal coatings.

## Experimental Section

### Materials

A waterborne latex dispersion was produced via a surfactant-free seeded emulsion polymerization reaction. The particles comprised poly(butyl acrylate-*co*-methyl methacrylate) (P(BA-*co*-MMA), 1:1 by weight) as well as 1 wbm % sodium styrene sulfonate (NaSS), which was used as an electrostatic stabilizer. A comprehensive report of the synthesis is provided elsewhere.^22^ Latex particles containing ionically bound Rhodamine B (RhB) were synthesized via seeded emulsion polymerization, as described in the SI.

**Figure 1:**
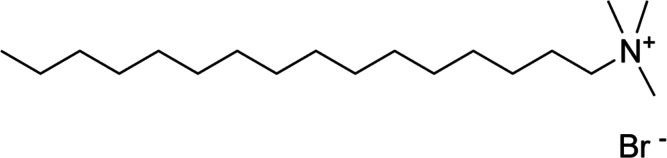
Chemical structure of cetrimonium bromide (CTAB) surfactant.

LUDOX TMA aqueous silica nanoparticle dispersion was obtained from Sigma-Aldrich and was used as received. The particles are modified with negatively charged aluminate groups to improve their stability in aqueous dispersion. Cetrimonium bromide (CTAB) surfactant was also obtained from Sigma-Aldrich (see Figure 1). Fluoresbrite YG microspheres (200 nm) were obtained from Polysciences.

The average hydrodynamic particle diameters of the latex, fluorescent latex, and silica nanoparticles were 246, 254, and 33 nm, respectively (determined by dynamic light scattering, see Figure S1). The polydispersity index values were calculated to be 0.02 (latex), 0.03 (RhB latex), and 0.10 (silica) using the formula PDI = (σ/*d*)^2^, where *d* is the mean diameter and σ is the standard deviation.^23^ All three particles were negatively charged, with zeta potential values of −58.4 ± 1.3 mV (latex), −59.3 ± 0.5 mV (RhB latex), and −26.7 ± 0.6 mV (silica). The zeta potentials were measured using electrophoretic light scattering. Values of less than −30 mV indicate that the latex particles were stable.^24,25^ The silica nanoparticle dispersion was less stable than desired and was therefore sonicated prior to use in experiments.

### Latex Film Preparation

Composite latex films containing silica and CTAB were prepared by casting and drying colloidal dispersion on glass substrates. The latex and silica were first diluted to 10 wt % solids content using deionized water. Before mixing, the silica dispersion was sonicated for 10 min to minimize aggregation. CTAB surfactant was added to the latex at several different concentrations between 0 and 1.5 wbm % (herein described as just %). These latex/CTAB dispersions were vortexed for 15 s, sonicated for 1 min, and vortexed again for 1 min to ensure adequate mixing. The silica dispersion was then added to obtain a silica volume fraction (in the initial wet films) of 0.013 as per our previous experiments^7^ (with a latex volume fraction of 0.062). The resulting silica volume fraction of the dried films was 0.17. When calculating these volume fractions, a density of 2.65 gcm^–3^ was used for the silica, while the density of the latex was estimated by taking the mean value between the densities of P(BA) and P(MMA) to give a value of 1.13 gcm^–3^. Fluorescent films were mixed using the same method but replacing the standard latex with the fluorescent latex particles. Samples were then coated with a dispersion of Fluoresbrite YG microspheres so that the top surface would be clearly visible during confocal fluorescence microscopy measurements.

The dispersions were then cast onto square glass coverslips (18 mm × 18 mm) that had been previously treated with an Ossila UV ozone cleaner for 10 min. Slightly larger coverslips (24 mm × 24 mm) were needed for fluorescence microscopy due to sample holder constraints. As per our previous studies, the dispersions were cast at volumes of 400 μL, though 200 μL was used for fluorescence microscopy samples to improve transparency and allow imaging through the entire thickness of the samples. The dispersions were then allowed to dry either at room temperature (21 ± 1 °C, 50 ± 5% relative humidity, RH) or under a high-humidity environment (>90% RH), to provide two different evaporation rates. The high-humidity environment was achieved by heating deionized water at 50 °C within a semi-sealed perspex container. The fast (room temperature) and slow (high humidity) evaporation rates were estimated at 1.4 × 10^–7^ m s^–1^ and 3.2 × 10^–9^ m s^–1^, respectively. These estimates were taken from previous literature involving similar drying conditions.^7,26^ Using the calculation method elaborated in the SI, the Péclet numbers (*Pe*) for latex and silica particles during drying at room temperature were calculated to be 75.4 and 10.1, respectively. With these values, and the silica volume fraction (ϕ_S_ = 0.17), small-on-top stratification is predicted by the model of Zhou et al., with the boundary condition α^2^(1 + *Pe*_S_) ϕ_S_ > 1 being satisfied^27^ (α is the size ratio between the particles). This has been observed in previous literature using very similar particles.^7^ The Péclet numbers of the particles during drying under high humidity were 2.2 (latex) and 0.3 (silica). While the value for silica was less than one, the conditions for stratification as defined by the ZJD model are still met, and the dispersions have also been shown to stratify.^7^

### Atomic Force Microscopy (AFM)

AFM topography images were obtained with a Bruker BioScope Resolve. Measurements were performed using tapping mode and silicon cantilevers (RTESPA-150) with typical spring constants of 5 N/m and tip radius of 8 nm. Images were analyzed using NanoScope Analysis 2.0 software.

### Confocal Fluorescence Microscopy

Confocal fluorescence microscopy was then conducted using a PicoQuant MicroTime 200 inverse time-resolved confocal microscope installed on an Olympus IX73. Samples were excited using a 482 nm diode laser. A UPLSAPO60XW Olympus objective lens mounted on a piezo and a 50 μm pinhole were used for imaging. Light emissions from the samples were then detected using a hybrid photomultiplier detector assembly (PMA). Confocal fluorescence microscopy images were processed using SymPhoTime (by PicoQuant) and ImageJ. Images were acquired in the *x-z* plane such that the cross section was observed. The image size was 30 μm × 30 μm, and resolution was 256 pixels × 256 pixels.

### Energy-Dispersive X-ray Spectroscopy (EDX)

Cross-sectional images of chemical composition were acquired using a JEOL JJSM-7800F FEG-SEM (field emission gun scanning electron microscope). Samples were prepared on standard microscope slides to allow fracturing without shattering (which occurred for glass coverslips). A diamond scribe was used to score the back of the slide, which was then snapped via freeze-fracture using liquid nitrogen. Samples were then placed on vertical SEM stubs and were coated with a gold/palladium alloy to improve conductivity. Relatively low accelerating voltages (5 keV) were used to minimize charge accumulation and sample damage.

### Light Scattering

A Malvern Zetasizer Ultra was employed to carry out both dynamic and electrophoretic light scattering (DLS and ELS) studies. Multiangle DLS was used to measure the average particle size of latex particles in a mixture containing silica and varied concentrations of the surfactant. This allowed us to analyze whether different particles were aggregating, leading to larger particle sizes. The dispersions containing silica nanoparticles and CTAB surfactant were prepared as before when producing films, although the silica/latex weight ratio was increased to 8:1 such that a peak for the silica particle size could be observed, as seen in Figure S2. Dispersions were diluted to 1 wt % with DI water before the measurement.

ELS was carried out using Malvern DTS070 folded capillary cells to measure the change in zeta potential of the latex particles as the concentration of surfactant was increased. CTAB surfactant was added to the latex dispersions, which were then diluted to 0.1 wt % with DI water. The same technique was attempted for dispersions containing silica nanoparticles; however, even at low surfactant concentrations, the dispersions underwent severe aggregation, making ELS measurements impossible. As explained by Wong et al.,^28^ the bonding of surfactants onto particles can cause them to become hydrophobic, resulting in destabilization and aggregation. For both particle size and zeta potential, mean averages were taken from three measurements for each sample.

### Ultraviolet–Visible Spectroscopy (UV–Vis)

The transparency of the latex films was assessed by carrying out visible light transmission studies using an Agilent Cary 5000 UV–Vis-NIR spectrophotometer with tungsten halogen visible and deuterium arc UV light sources. A background reading was used using an uncoated glass coverslip. This was then subtracted from subsequent measurements. The average transmission was calculated by taking the mean of transmission values across the visible range of wavelengths (400–750 nm). The wavelength step size was 1 nm.

## Simulation Section

### Langevin Equations

We consider first a single particle with radius *R*_1_ suspended in a fluid, where the molecules of the solvent have a radius *R*_2_. If *R*_1_ ≫ *R*_2_, it can be assumed that the large particles are subjected to a very large number of collisions with the solvent particles. This can be expressed in the form of a random force **ξ**(*t*), described hereafter. Another major contribution is the friction force, which, for low Reynolds numbers (laminar flow), can be assumed to be proportional to the velocity of the particle. All of the forces acting on the large particle can be written as
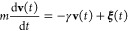
1In the case of a viscous fluid, the drag coefficient can be obtained from the equation
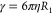
2where η is the viscosity of the fluid. The average of the noise term is zero for all components, i.e., ⟨ξ_*x*_⟩ = ⟨ξ_*y*_⟩ = ⟨ξ_*z*_⟩ = 0. The time correlation of the noise term can be expressed as ⟨ξ_*i*_(*t*)ξ_*j*_(*t*′)⟩ = 2*D*δ_*ij*_δ(*t* – *t*′) for *i* = *x*,*y*,*z*, where *D* is the bare diffusion constant, and δ is the so-called Dirac δ function. eq 1 can be rearranged to
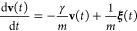
3which is the well-known Langevin equation^29^ for a single particle. When discussing  particles, additional contributions due to the interactions must be considered. These lead to  coupled equations of the form
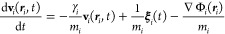
4where ***r***_*i*_ defines the position coordinate of the particle *i* and *m*_*i*_ is the mass. The force terms −∇Φ_*i*_ can be expressed as a function of the interparticle interaction potentials. The total potential Φ_*i*_ is generally defined as the sum of all contributions in the system, i.e.,
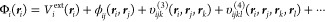
5where *V*_*i*_^ext^(*r*_*i*_) is the one-body external potential (e.g., gravitational field or potential due to the container walls), ϕ_*ij*_ describes the interactions between pairs of particles, υ_*ijk*_^(3)^ between triplets, and so on. It is a standard procedure to only account for pair contributions. Note that for identical particles, some indices can be dropped. The last equation then becomes
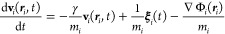
6

### Overdamped Equations of Motion

In an overdamped regime, the Langevin equation reduces to
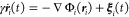
7where ***ṙ***_*i*_ represents the time derivative of the particle position. In this last formulation, the inertia of the particles is neglected. This is equivalent to assuming that the velocity–velocity correlation time scale τ_vv*i*_ = *m*_*i*_/γ is much smaller than the diffusion time scale τ (defined below). This last equation is a first-order stochastic differential equation, which can be solved using, for example, the Euler algorithm.^30^ This approach for molecular simulation is called Brownian dynamics. Since
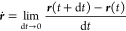
8one can obtain the trajectories of the particles by recursively solving
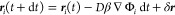
9where *D* = *k*_B_*T*/γ, β *=* 1/*k*_B_*T*, δ***r*** is the random displacement vector with each component sampled from a Gaussian distribution with standard deviation , and d*t* corresponds to the integration time step, and *k*_B_ is the Boltzmann constant and *T* is the temperature.

### Numerical Simulations

We perform Brownian dynamics simulations of a binary mixture composed by  big (b) and  small (s) particles. In other words, we solve  coupled Langevin equations in the overdamped limit, i.e., eq 7. We randomly initialized the particles in a box with size *L*_*x*_ × *L*_*y*_ × *L*_*z*_ = 10*R*_b_ × 10*R*_b_ × 15*R*_b_, where *R*_b_ is the radius of the b particles, without overlap.^30^ The random contributions used to generate the thermal Brownian motion of particles *i* = b,s are sampled from a Gaussian distribution with standard deviation , where *D*_*i*_ is the bare diffusion coefficient of the particles of species *i*. The system is confined between two parallel walls (both perpendicular to the *z*-axis). The lower of these walls models the surface onto which the colloidal mixture is deposited, and the upper wall models the influence of the water–air interface on the colloids. In the *x* and *y* directions, we use periodic boundary conditions. The upper wall descends in time from the position *z*_0_(0) = 15*R*_b_ to *z*_0_(*t*_f_) = 7.5*R*_b_, with constant velocity over the time interval *t*_f_ = 10τ, where the time unit τ is the big-particle Brownian time scale, τ = *R*_b_^2^/*D*_b_. This process is used for modeling solvent water evaporation.^10,12,31^ The interaction between particles of the same species is modeled via a truncated 12-6 Lennard-Jones (LJ) potential of the form
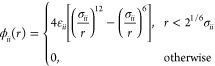
10where ε_*ii*_ defines the attraction strength between particles, and σ_*ii*_ = 2*R*_*i*_. Note, the cutoff means that ϕ_*ii*_(*r*) are purely repulsive. On the other hand, the cross-species interaction is modeled via a steep 56-28 LJ potential, i.e.
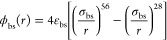
11where σ_bs_ = (*R*_b_ + *R*_s_)/2. In this work, we study two cases. Case 1 simulates a scenario where particles are modeled as hard spheres with no strong electrostatic interactions. We consider only the repulsive part of the potential in eq 11, meaning that we cut off this interaction at the minimum of the potential, i.e., at *r* = 2^1/28^σ_bs_. For this case, we set ε_bs_ = *k*_B_*T*. In Case 2, the interaction between the big and small particles is attractive. We thus truncate the potential in eq 11 at at *r* = 1.5σ_bs_ (note that ϕ_bs_(1.5σ_bs_) ≈ −5 × 10^–5^ε_bs_) and set ε_bs_ = 10*k*_B_*T*. Simulations were also carried out with ε_bs_ set to 5*k*_B_*T* and 7.5*k*_B_*T* to provide a range of interaction strengths.

The lower wall exerts a repulsive force on the suspended particles of the form  if |*z* – *z*_0_| < σ_*i*_^w^, and *F*(*z*) = 0 otherwise, where σ_*i*_^w^ defines the softness of the wall and *z*_0_ is the position of the surface of the wall and is set to σ_b_^w^ = *R*_b_ + *R*_s_ and σ_s_^w^ = 2*R*_s_, respectively. The upper wall exerts the same force but in the opposite direction. The following additional parameters have been used for both cases: ϵ_ss_ = ϵ_bb_ = *k*_B_*T*, d*t* = 10^–6^τ,  = 50,  = 7500, *R*_s_ = *R*_b_/8, and *D*_s_ = *D*_b_*R*_b_/*R*_s_.

## Results and Discussion

To investigate the effects of particle interactions on colloidal self-assembly, we introduce a cationic surfactant (CTAB) to binary latex/silica dispersions that are known to stratify upon drying and to form silica superstructures.^7^ We analyze the effect of surfactant concentration on the stratification and the final film morphology of the colloidal films using AFM, EDX, and confocal fluorescence microscopy. In addition, we have Brownian dynamics simulations that, with the above choices of interactions and evaporation (wall-moving) rates, agree with the experimental results. Our experiments and computer simulations thus together lead to a clear understanding of how particle interactions lead to the observed dried-on structures.

**Figure 2:**
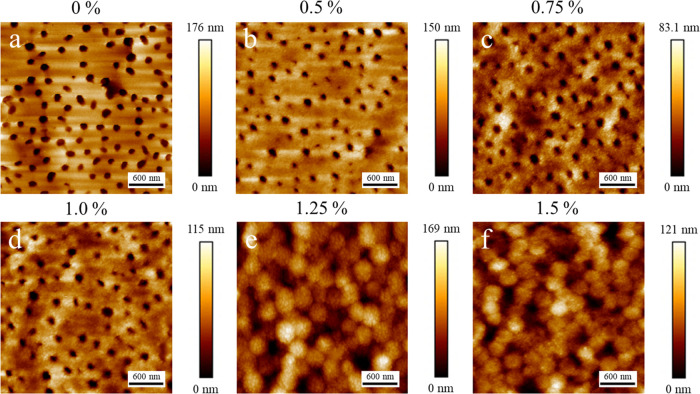
AFM topography images of binary latex/silica films containing different concentrations of CTAB surfactant (as indicated above each) dried at room temperature (21 ± 1 °C, 50 ± 5% RH). Image sizes are 3 μm × 3 μm.

Figure 2a shows the AFM topography image of a binary latex/silica film containing no surfactant, dried at room temperature. Within the image, it can be seen that the top surface comprises a layer of silica nanoparticles. There are holes in the silica structure, at the bottom of which are latex particles (as shown by our previously reported elastic modulus maps^7^). The spacing between the holes is of a similar size to the latex particles, indicating that the silica superstructure is templated in some way by the latex structure below. Previously, we explained the formation of the structures by showing that there is an effective repulsive interaction between the negatively charged latex and silica particles and that this repulsion is larger than that experienced between the silica particles. This causes the silica particles to build up in the free spaces between the latex particles rather than forming a homogeneous coating. By introducing cationic surfactants, we must expect the magnitude of the negative charge on the latex particles to be reduced, resulting in a weaker repulsive interaction between particles. Given our previous observations and explanations,^7^ we should perhaps expect to observe more homogeneous silica layers at the film surfaces as the surfactant is added.

Initially, the addition of the surfactant has little effect on the surface morphology of the films. Figure 2 shows that the characteristic silica superstructures persist up to a surfactant concentration of 1.0%, with the only difference being the height of the silica layers. As seen in Figure S3 of the Supporting Information, the average height of the silica superstructures reduces significantly (by 35 nm) as the surfactant concentration increases from 0 to 1.0%.

**Figure 3:**
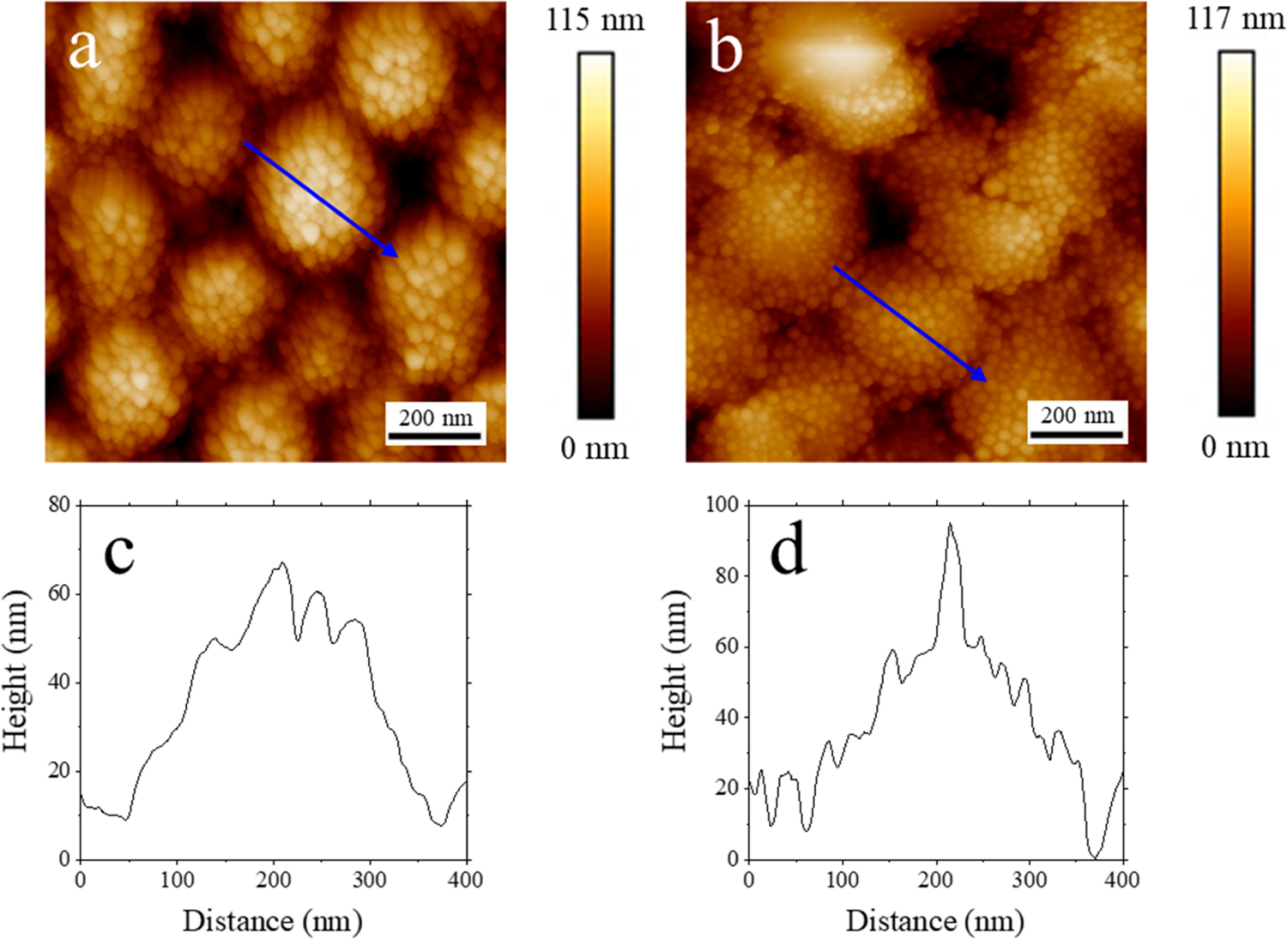
AFM topography images of size 1 μm × 1 μm of binary latex/silica film containing 1.5% CTAB surfactant dried at (a) room temperature and (b) high humidity. Panels (c) and (d) show examples of plots of the topography height along the cross sections indicated by the blue lines in the AFM images.

Once the surfactant concentration is increased to 1.25%, the surface morphology of the films changes entirely. In Figure 2e–f, a latex particle-like morphology is observed. When the AFM image size is reduced to 1 μm × 1 μm (see Figure 3a), it is clear that the surface still comprises silica particles. The silica layer now completely covers the latex. As shown by AFM height cross sections, in Figure 3c,d, these larger circular patterns are of a similar magnitude of size to the latex particles. Furthermore, the average size of the large circular patterns within the AFM image was calculated using ImageJ and was equal to 305 ± 20 nm. The size of one latex particle (250 nm) plus two silica particles (40 nm) equals 290 nm. This value falls within the range of uncertainty of the average measured by AFM. It, therefore, seems that the final surface morphology is a monolayer of silica particles lying above the bulk latex, and that is why the overall texture of the films when viewed at lower magnifications appears similar to that of a monodisperse latex film.

The most likely scenario that could lead to the formation of these surface morphologies is that the latex particles become fully coated in silica nanoparticles prior to drying, referred to as armored particles. The addition of surfactants may counteract the repulsion between latex and silica particles. If electrostatic repulsion is not enough to counteract the van der Waals forces, then they may form armored latex particles during mixing and drying. If the silica particles are anchored to the latex particles, then stratification will not occur. However, the surface of the final dried structure will still largely be formed of silica particles since they coat the latex particles.

Although unlikely, it is also possible that stratification is still occurring to a degree and that the homogeneous coverage is due to the reduction in repulsive particle interactions, allowing the silica particles to fully coat the latex. Given the prominence of the circular patterned texture caused by the large latex particles below the surface, it appears that the silica layer at the surface has a much lower height when compared with films containing no surfactant. This would mean that there is more silica in the bulk of the film, caused by aggregation of the silica particles preventing higher degrees of stratification.

**Figure 4:**
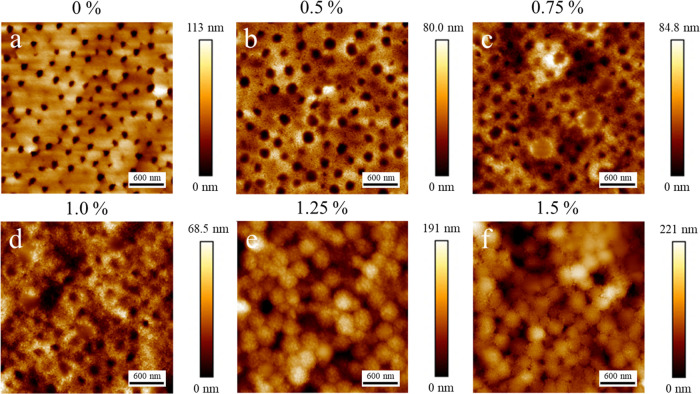
AFM topography images of binary latex/silica films containing different concentrations of CTAB surfactant (as indicated above each), dried under high humidity (21 ± 1 °C, >90% RH). Image sizes are 3 μm × 3 μm.

Binary latex/silica dispersions containing surfactant were also dried under high humidity to investigate the effects of a reduced Péclet number. Very similar results can be seen as before with films dried at room temperature. Figure 4 shows that up to a surfactant concentration of 1.0%, there is little change in the surface morphology, though there is a reduction in the height of the silica layer similar to samples dried at room temperature (decreased by 36 nm, as seen in Figure S3). The superstructure heights in samples dried under high humidity are lower than in those dried at room temperature. This is likely due to weaker stratification effects at lower evaporation rates, as suggested previously.^7^ As before, at surfactant concentrations of 1.25 and 1.5%, the morphology changes entirely, displaying a latex-like texture when observed in larger images (3 μm × 3 μm), as seen in Figure 4e–f. However, at higher magnification (see Figure 3b), the surface clearly still contains only silica particles.

These results support the theory that armored particles form in wet dispersion. If the structures were caused by stratification but with some aggregation leading to depleted silica surface layers, then the CTAB concentration at which the transition occurs would likely be more dependent on evaporation rates. At lower evaporation rates, larger degrees of aggregation would be expected, as there is more time for the aggregates to form. The transition between the two morphologies would then likely be expected at lower surfactant concentrations.

**Figure 5:**
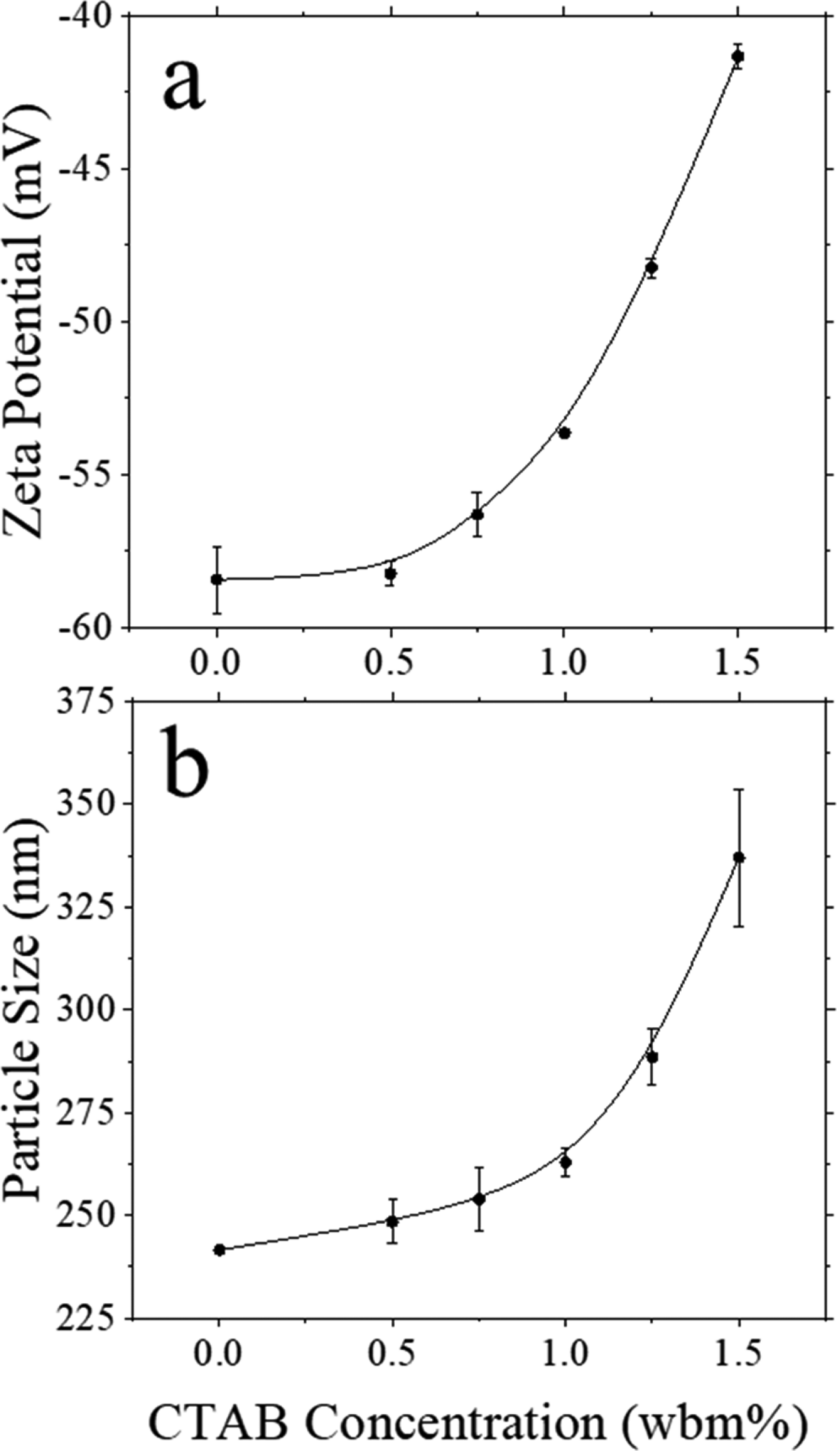
(a) Zeta potentials of the latex dispersion containing different concentrations of CTAB surfactant, measured by ELS. (b) Average particle size within a binary latex/silica dispersion (1:8 by weight) containing different concentrations of CTAB surfactant.

DLS and ELS were utilized to provide further evidence as to which of the two theories mentioned earlier more accurately describes the assembly mechanisms. The zeta potential was measured for the latex dispersion as the surfactant concentration was increased. As expected, the zeta potential becomes less negative with the surfactant concentration, as seen in Figure 5a. The change is slow at first, only increasing from −58.4 mV to −53.6 mV at a concentration of 1.0%. After this point, the increase is slightly more rapid, ending with a final zeta potential of −41.3 mV. While the latex particles’ zeta potential is changed by the presence of the surfactant, it does not come close to becoming overall positively charged. This might possibly suggest that armored particles are unlikely since the negatively charged (−26.7 mV) silica particles will still be repulsed by the latex. However, it is possible that the surfactant also bonds to the silica particles. This could sufficiently reduce the repulsion between the particles such that the van der Waals forces destabilize them, leading to the formation of armored particles. Depletion interactions due to the presence of CTAB could also promote the formation of armored particles; however, this is probably unlikely in the dilute systems we characterized by DLS.^32^ In more concentrated dispersions, such as those used for drying experiments, the presence of less water would likely favor adsorption of surfactants to the surface of the particles, which would make the formation of armored particles even more likely. We were unable to measure the zeta potential values for silica dispersion containing surfactant due to aggregation, suggesting a strong affinity between the surfactant and silica and a possible significant reduction in surface charge.

Looking at the average size of the particles in the system paints a different picture. The surfactant concentration was varied in dispersions containing both latex and silica particles, and the average size of the (latex) particles was measured via DLS. The graph shown in Figure 5b shows that the average particle size increases with surfactant concentration. The exact size of the increase in average latex particle diameter strongly supports the idea that armored particles are being formed. The particle size does not come close to doubling, meaning that it must be the smaller silica particles attaching to the latex particles and causing the increase as opposed to latex particles aggregating together. In particular, at a surfactant concentration of 1.25%, the average particle diameter is equal to the size of one latex particle (250 nm) plus two silica particles (40 nm). Plots of the correlation function vs time, as shown in Figure S4, also indicate that the increased particle size is due to armored particle growth rather than a random distribution of aggregates.

**Figure 6:**
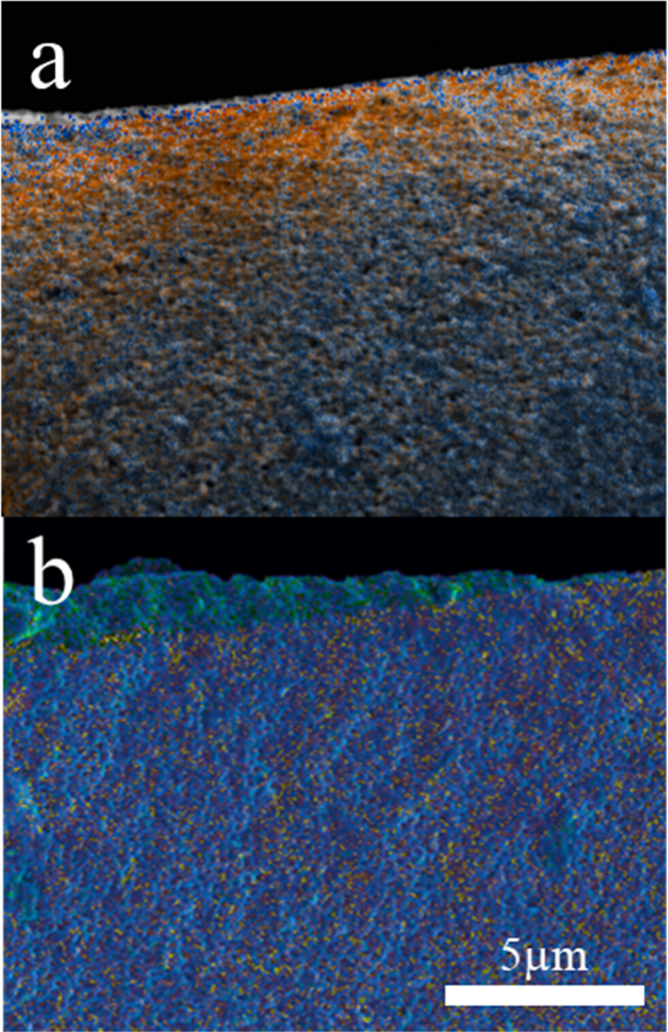
Cross-sectional EDX maps of binary latex/silica films containing (a) 0% and (b) 1.5% CTAB surfactant. Silicon and carbon are shown in orange and blue, respectively.

To shed light on the particle distribution within the dried films, we produced cross-sectional images using EDX and confocal fluorescence microscopy. Figure 6a shows the EDX cross section of a binary latex/silica film containing no surfactant. Silicon (shown in orange) is very prominent at the surface and decreases in intensity further down, being replaced by the blue of carbon. This clearly indicates that the film is enriched in silica nanoparticles near the top surface. Such gradient in intensity is not observed in films with higher surfactant levels (1.5%), as seen in Figure 6b, indicative of a homogeneous distribution of silica particles throughout the film thickness.

**Figure 7:**
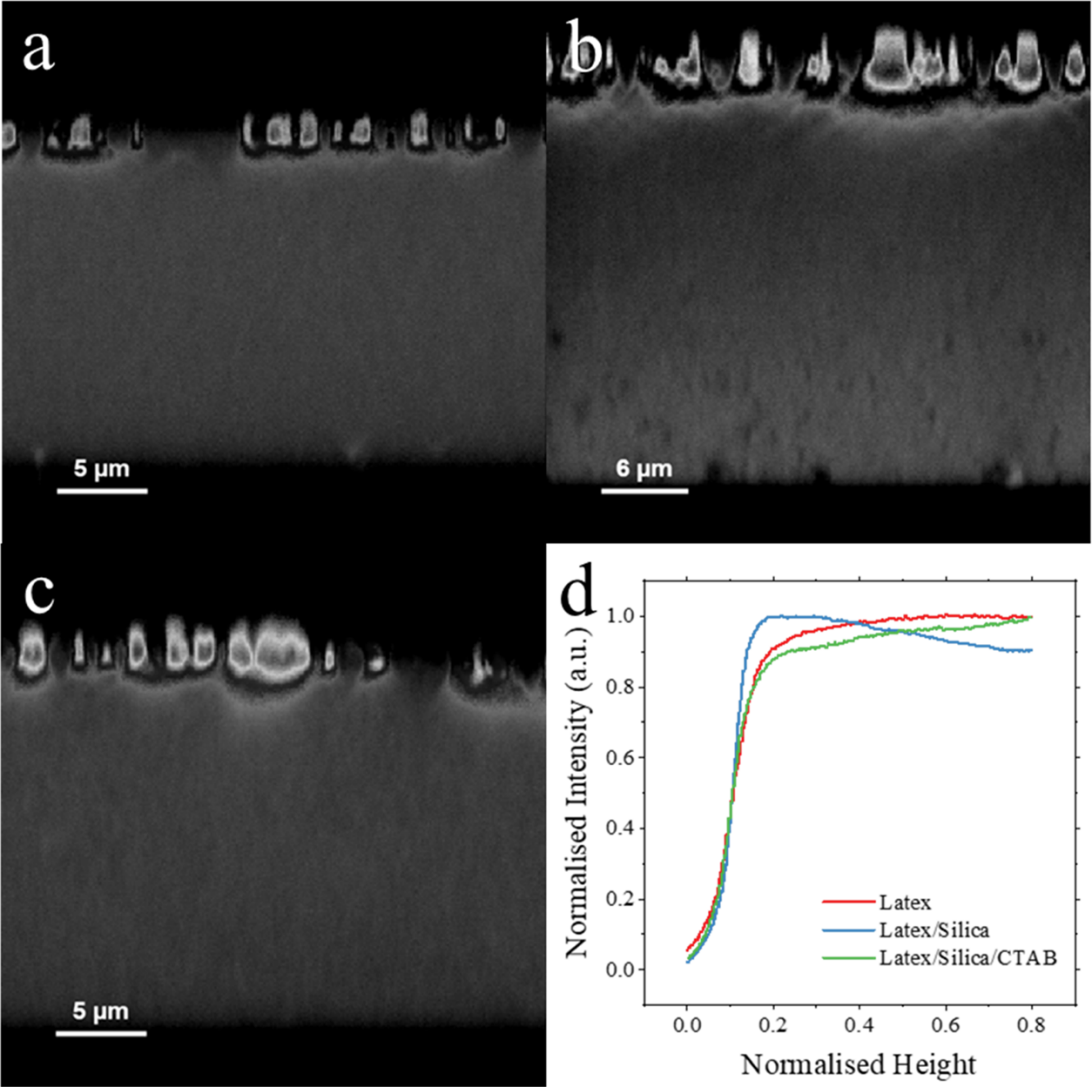
Confocal fluorescence microscopy cross-sectional images of (a) a control latex film, (b) a binary latex/silica film, and (c) a binary latex/silica film containing 1.5% CTAB surfactant. (d) Graph showing the average intensity profiles along the film height for each sample. Relative intensity was calculated by dividing by the maximum intensity for each image, normalized height was calculated by dividing by the total film height. The profiles are cut off at the top surface of the film, which was located using the coating of Fluoresbrite YG microspheres.

Similar trends can be seen in the analysis of cross-sectional images created by confocal fluorescence microscopy, as shown in Figure 7a–c. Here, latex particles have fluorescent tags, and films are coated with strongly fluorescing Fluoresbrite YG microspheres. Therefore, stratified films will have a layer of nonfluorescing silica particles. A gradient in intensity of fluorescent light can be seen in binary latex/silica films without surfactant, which can be seen more clearly in the intensity profile, as shown in Figure 7d. Toward the surface of the film, the intensity decreases due to the enrichment in silica particles. The intensity gradient is not observed in either the control latex or films containing surfactant, indicating the lack of a stratified silica layer.

To provide further evidence on the likelihood of the formation of armored particles, we consider a geometric model of the system to calculate the volume of silica nanoparticles that would be required to coat the latex. First, we calculate the difference between the volumes of an armored particle with a diameter of 290 nm (volume of 1.0 × 10^8^ nm^3^) and an uncoated latex particle with a diameter of 250 nm (6.5 × 10^7^ nm^3^) to give a shell volume of 3.6 × 10^7^ nm^3^. This suggests that the volume fraction of the shell compared with the core of the particle is 0.36, which should therefore be the optimum silica volume fraction in the film for full coverage to occur. However, this simple geometric model does not account for void spaces. The packing factor of a three-dimensional hexagonal close-packed structure is only 74%, meaning that the optimum silica volume fraction is reduced to 0.27. Also, the silica particles are unlikely to form a completely dense shell (close-packing may not be achieved). Therefore, despite the silica volume fraction (0.17) being below this optimum value, it seems likely that there is a high-enough silica content for the latex particles to be significantly coated. In addition, particles at the surface of the film will likely appear to be more coated than those below, since residual free silica particles are likely to be trapped by the descending liquid–air interface, enriching the top surface with silica.

**Figure 8:**
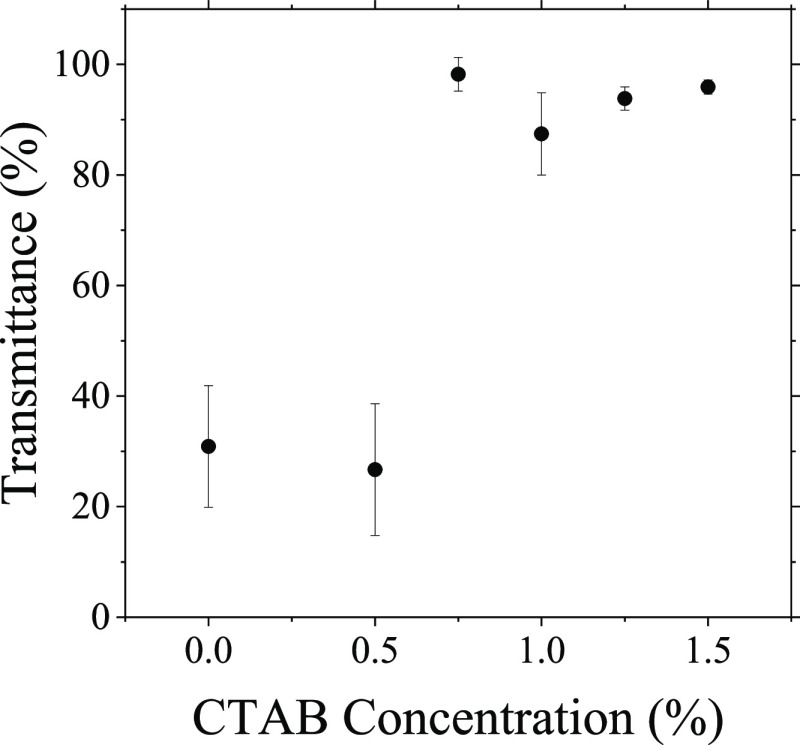
The average light transmittance (as a percentage) through binary latex/silica films containing different surfactant concentrations (dried at room temperature) as measured by UV–Vis spectroscopy.

It is also interesting to note the increase in optical transparency of the films as the surfactant concentration is increased. This can be seen visually; however, UV–Vis spectroscopy was used to quantify the exact percentage transmittance of light through the films. The results are shown in Figure 8. The standard latex/silica films without surfactant are relatively opaque, with transmittance values of around 30%. This provides more evidence that there is a stratified layer of silica nanoparticles at the surface of the film. The silica nanoparticles are hard and do not deform during drying. A thicker layer of these particles makes scattering more likely, preventing light from passing straight through, and resulting in a more opaque film. In samples containing the surfactant, the silica particles are spread more homogeneously throughout the film height, making light scattering less likely and resulting in more transparent films, evident from transmittance values close to 100%. This increase in transmittance occurs at surfactant concentrations of 0.75% and above, indicating that the height of the silica layer is, in fact, reduced, and silica nanoparticles are more homogeneously distributed in *z*. This is in agreement with the AFM images shown earlier.

Altogether, our results indicate that the addition of surfactant results in the formation of armored particles, with the latex particles being coated in silica nanoparticles. This leads to a transition toward a more homogeneous silica coverage; however, the stratification effects are also suppressed. To confirm whether these changes are a result of changing particle interactions, we modeled the system using Brownian dynamics simulations. We start with a randomly initialized mixture of large and small particles between two parallel interfaces. The drying is then simulated by a descending top interface, which is strongly repulsive to all particles. To model the system without surfactants (Case 1), we set the interaction between the large and small particles to be purely repulsive. We can be confident that this is the situation in the physical experiments as both particle types are negatively charged, as evident from their zeta potentials. Our experimental results suggest that there is an attraction between large and small particles once the surfactant is added. Therefore, we also simulate a situation (Case 2), where the large and small particles are subjected to attractive forces between each other.

**Figure 9:**
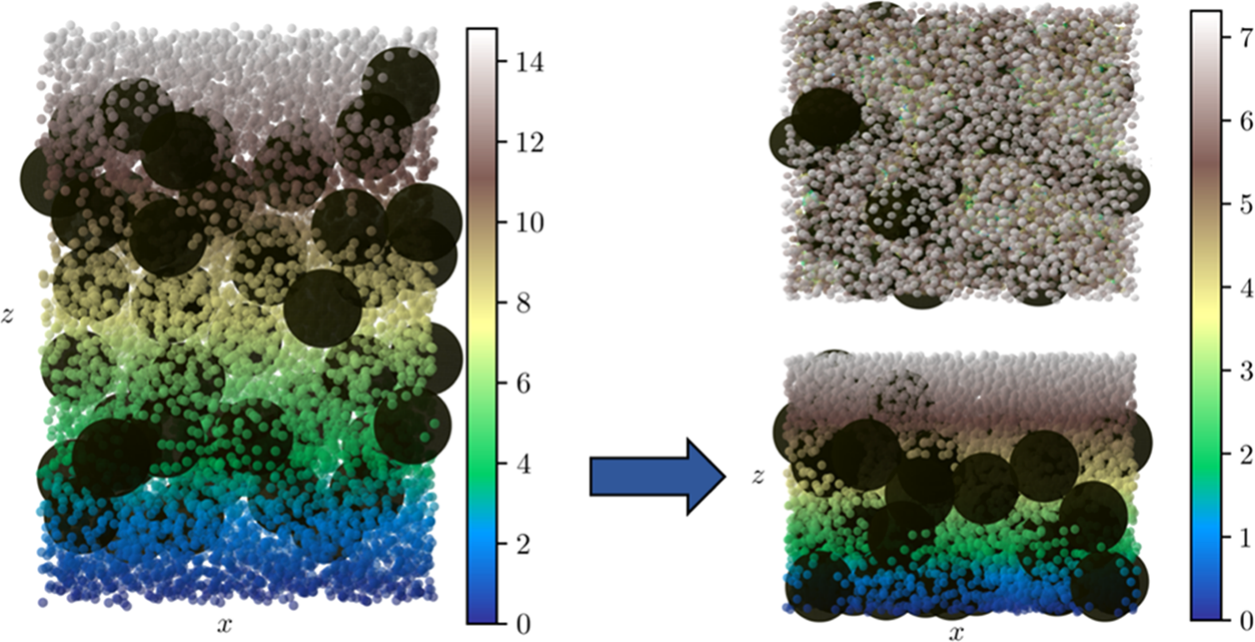
Results obtained from Case 1 (purely repulsive big–small interactions) before (left) and after (right) simulations. The top result on the right shows the top view of the final system, while the bottom result is the side (cross section) view. The color bar indicates the *z*-coordinate of the small particles in units of *R_b_*. The top view of the initial system can also be viewed in Figure S5.

Figure 9 shows the positions of all particles before and after carrying out the simulation of Case 1. After drying, it can be seen that the top surface is enriched with smaller particles, which form a relatively homogeneous layer. This matches the stratification effect that is observed experimentally when drying binary latex/silica films containing no surfactant. These results are also in agreement with previously accepted models of stratification such as those published by Fortini et al.^12^ and Zhou et al. (the ZJD model).^27^ They indicate that larger particles are more susceptible to diffusiophoresis compared with smaller particles, leading to small-on-top stratification.

**Figure 10:**
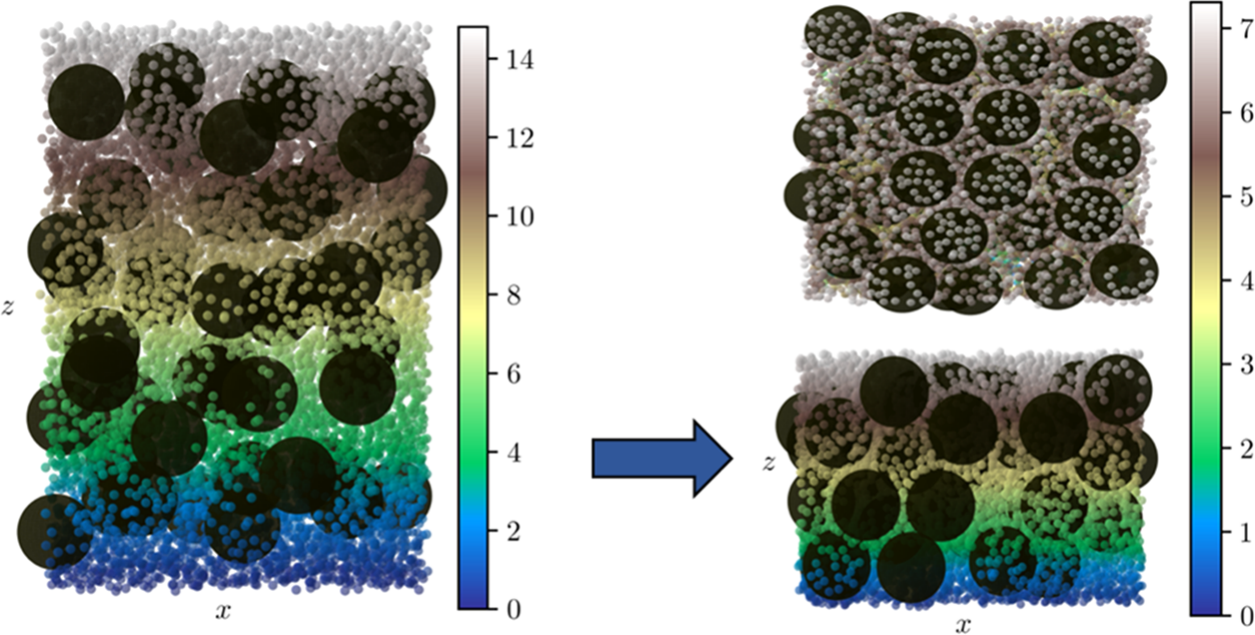
Results obtained from Case 2 before (left) and after (right) simulations. The top result shows the top view of the final system, while the bottom result is the front view. The color bar indicates the *z*-coordinate of the small particles in units of *R_b_*. The top view of the initial system can also be viewed in Figure S5.

Simulations of Case 2, where large and small particles are given an attractive interaction, show that stratification no longer occurs. It can be seen in Figure 10 that there is not a distinct layer of small particles at the top surface. Instead, there is a relatively homogeneous mixture of the two particles, with a significant amount of the small particles seemingly attached to the surface of the larger particles. This matches well with the experimental results, providing good evidence that the surfactants within the system do in fact result in effective, attractive interactions between the two different types of particles. Similar to the experiments, the simulation results also indicate that the change in interaction results in the formation of armored large particles coated with single layers of small particles. This effect can be seen to increase as the strength of attractive interactions is increased between the large and small particles. This can be seen in Figures S6 and S7, which show simulation results, where ε_bs_ is set to 5*k*_B_*T* and 7.5*k*_B_*T*, respectively.

## Conclusions

In this work, we have shown that particle interactions play an important role in deciding the final film architectures of drying binary colloidal systems. We started with a binary mixture of large latex and small silica particles known to stratify during drying from our previous work. Both species of particles were charge-stabilized with a negative charge, leading to repulsive interactions between each other. By imaging the surface topography of dried films using AFM and analyzing cross sections with EDX and fluorescence confocal microscopy, we have shown conclusively that a stratified layer enriched with silica nanoparticles forms during drying. We repeated these measurements for latex/silica mixtures containing a cationic surfactant to observe the effects of altering the particle surface charges and therefore the interactions between particles. We found that the stratification effect could be switched off entirely just by adding these surfactants.

By utilizing ELS to measure the particle zeta potentials, we showed that the surface charge of the large latex particles could be controlled by the surfactant concentration. We also showed, using DLS, that this modification in surface charge causes an attraction to the small silica particles, resulting in the formation of armored particles. This prevents the silica particles from having the freedom to separate during drying to form a stratified surface layer. To prove that this change comes about because of the change in particle interactions, we modeled the system using Brownian Dynamics simulations, both with attractive and repulsive forces between the two particle types. The simulations corroborated our results, showing that attractions between small and large particles prevented stratification, instead forming the same armored particles.

The work here shows how surfactants can be used to control the degree of stratification in colloidal films. Since stratification allows to add surface functionality, the present work provides a valuable tool for tuning the architecture and performance of functional coatings. Furthermore, although it was not the primary focus of this work, we have shown how stratification may actually be prevented where necessary using a simple method. This prevention has a significant effect on the transparency of composite latex films, which is a highly desirable property in silica/polymer nanocomposites.

## Supporting Information Available

Dynamic light scattering data for colloidal particles and for latex/silica/surfactant mixtures; latex synthesis method; Péclet number calculations; average superstructure heights (PDF)
