# Supplementary material for: Effect of Particle Interactions on the Assembly of Drying Colloidal Mixtures
Source: Langmuir. 2022 Apr 19;38(18):5361–71. doi: 10.1021/acs.langmuir.1c03144 (PMC9097527; doi:10.1021/acs.langmuir.1c03144)
Supplement: Supplementary file 1 — la1c03144_si_001.pdf [file la1c03144_si_001.pdf]

Supporting Information

Role of Particle Interactions on the Assembly of drying Colloidal Mixtures

*James D. Tinkler *[a], Alberto Scacchi [b,c], Maialen Argaiz [d], Radmila Tomovska [d,e], Andrew J. Archer [f], Helen Willcock [a], Ignacio Martín-Fabiani [a]*

[a] Department of Materials, Loughborough University, Loughborough, LE11 3TU, U.K.

[b] Department of Chemistry and Materials Science, Aalto University, P.O. Box 16100, FI-00076 Aalto, Finland

[c] Department of Applied Physics, Aalto University, P.O. Box 11000, FI-00076 Aalto, Finland

[d] POLYMAT and Departmento de Química Aplicada, Facultad de Ciencias Químicas, University of the Basque Country, UPV/EHU, Joxe Mari Korta Zentroa, Tolosa Hiribidea 72, Donostia-San Sebastian, 20018, Spain

[d] Ikerbasque, Basque Foundation for Science, Maria Diaz de Haro 3, 48013 Bilbao, Spain

[f] Department of Mathematical Sciences and Interdisciplinary Centre for Mathematical Modelling, Loughborough University, Loughborough, LE11 3TU, U.K.

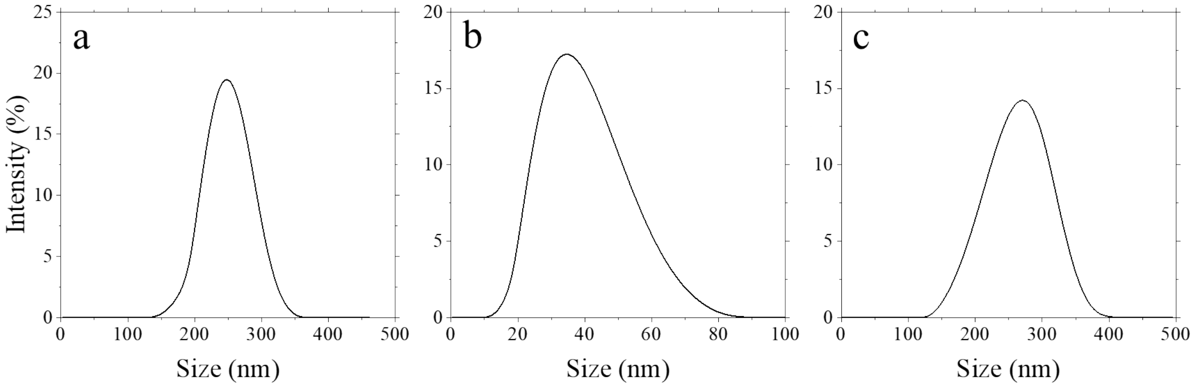

Figure S1. Particle size intensity distributions measured by dynamic light scattering (DLS) for (a) latex particles, (b) silica nanoparticles, and (c) fluorescent latex particles.

Fluorescent Latex Synthesis. Latex particles containing ionically bound Rhodamine B (RhB) were synthesised via a seeded emulsion polymerisation similar to that used to create the standard latex particles. Ascorbic acid/tert-butyl hydroperoxide (AsAc/TBHP) redox couple initiator was used. RhB fluorescent dye (0.4 wbm%) was dissolved in an aqueous solution containing sodium styrene sulfonate (NaSS) and AsAc (0.54 wbm%). This solution was fed into the seeded reaction mixture with the monomers (BA and MMA) and TBHP (0.54 wbm%). RhB incorporation was investigated by centrifuging the latex dispersion. It was clear after centrifuging that the separated latex particles were coloured. The supernatant was also analysed quantitatively using ultraviolet-visible (UV-Vis) spectroscopy. Reduced RhB signals observed in the UV-Vis indicated successful incorporation of RhB into the particles.

Péclet Number Calculations. Péclet numbers of colloidal particles were calculated using the formula:

$$Pe = \frac{6\pi\mu RH\dot{E}}{kT}$$

Where μ is the viscosity of water, R is the particle radius, H is the initial height of the film, $\dot{E}$ is the evaporation rate, k is the Boltzmann constant, and T is the temperature. As shown in Figure S1, the radius of the large latex and silica particles were 123 nm, and 16.5 nm, respectively. The initial film height was estimated at 1.2 mm by considering the total volume of dispersion and the surface area of the substrate. When drying at slow and moderate evaporation rates the temperature was 21 ± 1 °C (294 K), resulting in a water viscosity of 1×10^{-3} Pa s. Values for evaporation rate were taken from the work of Utgenannt *et al.*[1] due to the similarities in experimental conditions. Slow and moderate evaporation rates were 3.2×10^{-9} m/s and 1.1×10^{-7} m/s, respectively.

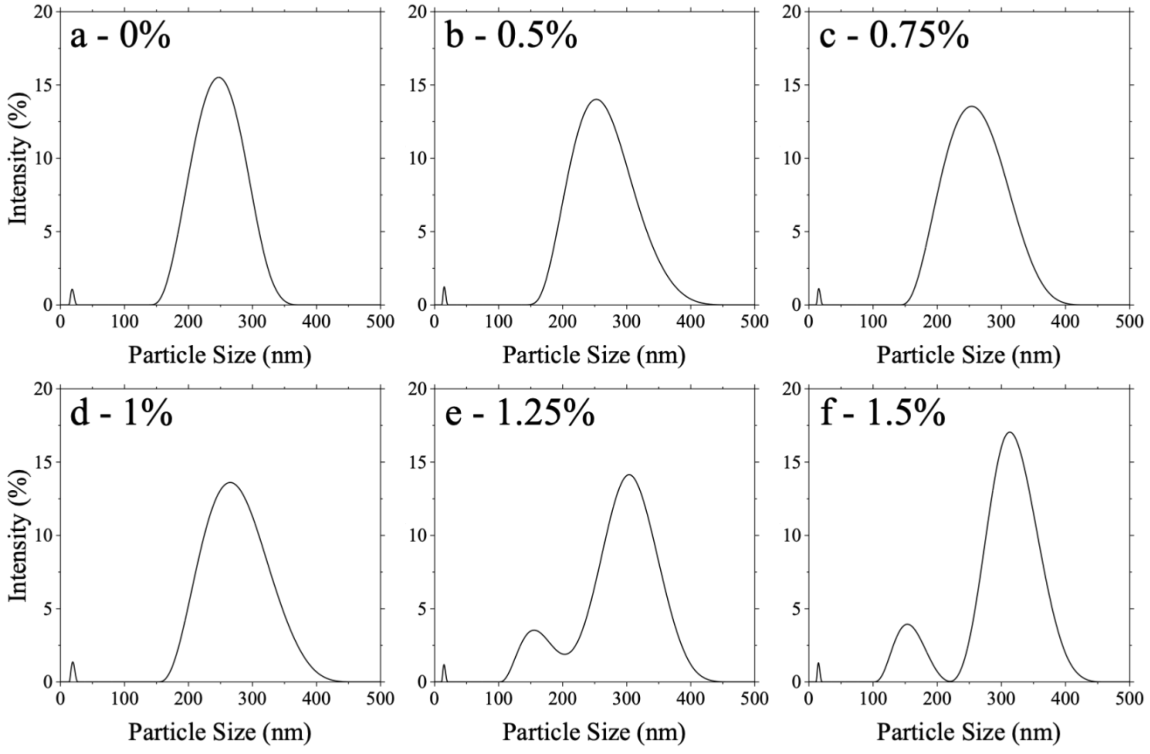

Figure S2. DLS particle size data for latex/silica dispersions (1:8 by weight) containing different CTAB surfactant concentrations.

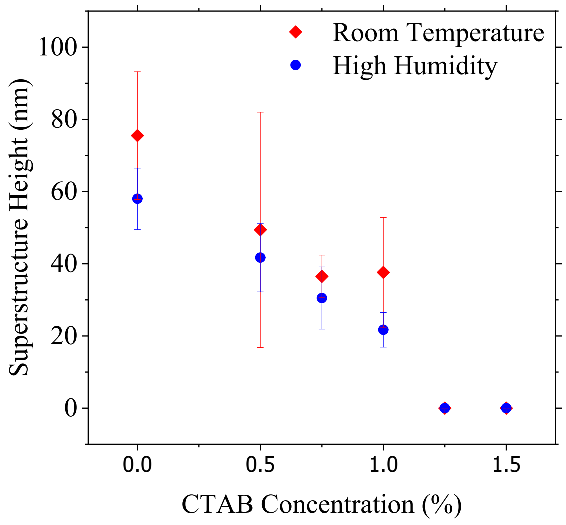

Figure S3. Average surface superstructure heights for latex/silica films containing different CTAB surfactant concentrations. We define the superstructure height as being the average height of the top surface above the height of the bottoms of the holes in which latex particles are located – compared from AFM data in Figures 1 and 2.

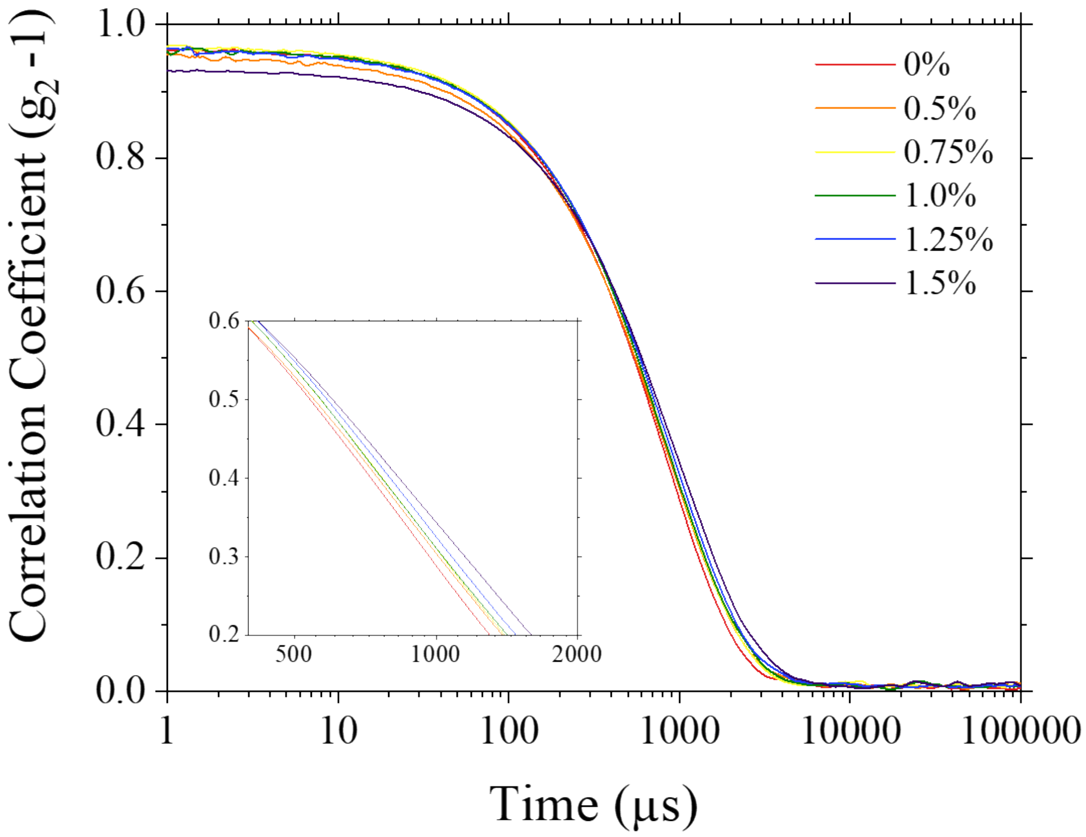

Figure S4. Correlation function vs time taken from DLS measurements of latex/silica dispersions containing varied surfactant concentration.

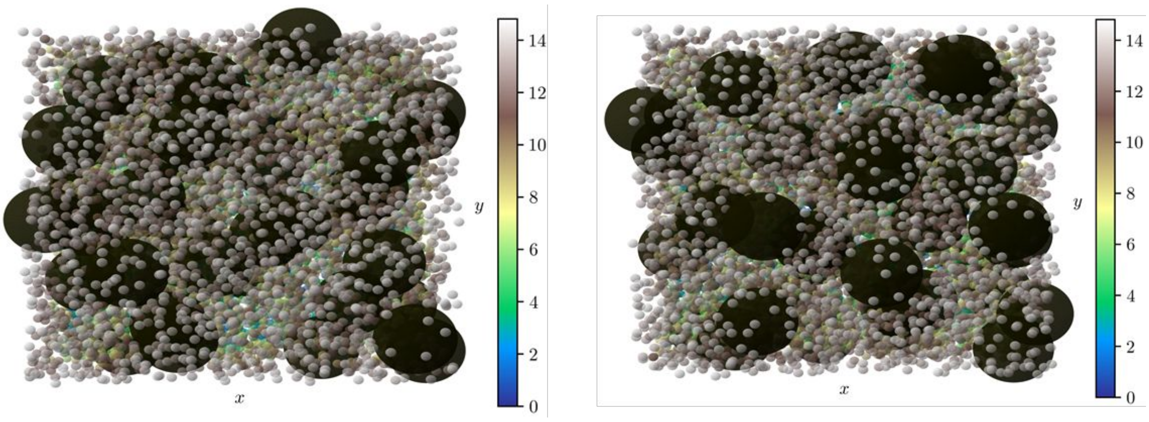

Figure S5. Top view of the initial systems before simulations for Case 1 (left) and Case 2 (right). The colour bar indicates the *z*-coordinate of the small particles in units of $\boldsymbol{R_b}$.

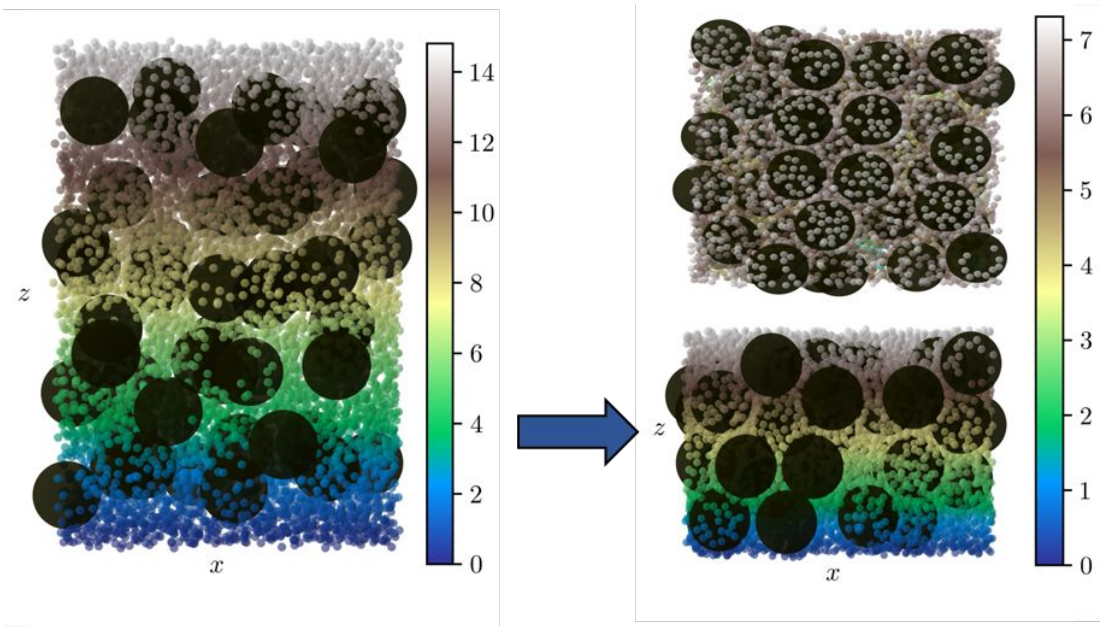

Figure S6. Simulation results obtained from Case 2 (with ε_{bs} set to $5k_BT$) before (left) and after (right) simulations. The top result shows the top view of the final system while the bottom result is the front view. The colour bar indicates the *z*-coordinate of the small particles in units of $\boldsymbol{R_b}$.

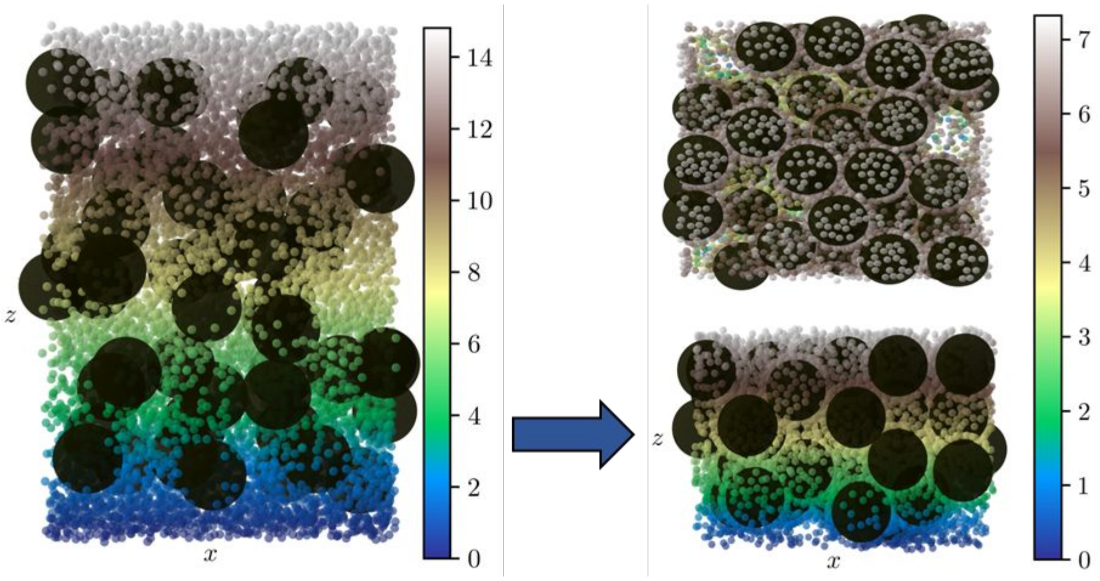

Figure S7. Simulation results obtained from Case 2 (with ε_{bs} set to $7.5k_BT$) before (left) and after (right) simulations. The top result shows the top view of the final system while the bottom result is the front view. The colour bar indicates the *z*-coordinate of the small particles in units of $\boldsymbol{R_b}$.

References

1. Utgenannt, A.; Maspero, R.; Fortini, A.; Turner, R.; Florescu, M.; Jeynes, C.; Kanaras, A. G.; Muskens, O. T.; Sear, R. P.; Keddie, J. L. Fast assembly of gold nanoparticles in large-area 2D nanogrids using a one-step near-infrared radiation-assisted evaporation process, ACS Nano, 2016, 10, 2232-2242
